# Distributions and determinants of urinary biomarkers of organophosphate pesticide exposure in a prospective Spanish birth cohort study

**DOI:** 10.1186/s12940-017-0255-z

**Published:** 2017-05-17

**Authors:** Sabrina Llop, Mario Murcia, Carmen Iñiguez, Marta Roca, Llúcia González, Vicent Yusà, Marisa Rebagliato, Ferran Ballester

**Affiliations:** 1 Epidemiology and Environmental Health Joint Research Unit, FISABIO−Universitat Jaume I−Universitat de València, Av. Catalunya 21, 46020 Valencia, Spain; 2 Spanish Consortium for Research on Epidemiology and Public Health (CIBERESP), Av. Monforte de Lemos, 3-5. Pabellón 11, 28029 Madrid, Spain; 3 Analytical Unit, Medical Research Institute Hospital La Fe, Av. Fernando Abril Martorell, 106- Torre A, 46026 Valencia, Spain; 4 Foundation for the Promotion of the Health and Biomedical Research in the Valencia Region, FISABIO-Public Health, Av. Catalunya 21, 46020 Valencia, Spain; 5 Public Health Laboratory of Valencia, Av. Catalunya 21, 46020 Valencia, Spain; 6 Analytical Chemistry Department, Universitat de València, Dr. Moliner, 50, 46100 Burjassot, Valencia Spain; 7 Department of Medicine, Universitat Jaume I, Av. Vicent Sos Baynat, s/n, 12071 Castelló de la Plana, Spain; 8 Foundation for the Promotion of Health and Biomedical Research in the Valencia Region, FISABIO-Public Health, Avda. Catalunya 21, 46020 Valencia, Spain

**Keywords:** Pesticides, Diet, Vegetables, Fruit, Agriculture, Development

## Abstract

**Background:**

Prenatal exposure to organophosphate pesticides (OPs) has been associated with impaired child development. Pesticide exposure determinants need to be studied in order to identify sources and pathways of pesticide exposure. The aim of this paper is to describe prenatal exposure to OPs and evaluate the associated factors in pregnant women.

**Methods:**

The study population consisted of pregnant women (*n* = 573) who participated in the INMA birth cohort study in Valencia (Spain, 2003–2006). OP metabolites were analyzed in maternal urine at the 32nd week of gestation using a liquid chromatography-high resolution mass spectrometry method. The analysis included non-specific (diethyl phosphate [DEP], diethyl thiophosphate [DETP], dimethyl thiophosphate [DMTP], dimethyl dithiophosphate [DMDTP]) and specific metabolites (2-diethylamino-6-methyl-4-pyrimidinol [DEAMPY], 2-isopropyl-4-methyl-6-hydroxypyrimidine [IMPY], para-nitrophenol [PNP], and 3,5,6-trichloro-2-pyridinol [TCPY]). Information about the sociodemographic, environmental, and dietary characteristics was obtained by questionnaire. The association between log-transformed OPs and covariates was analyzed using multivariable interval censored regression.

**Results:**

The detection frequencies were low, DMTP and TCPY being the most frequently detected metabolites (53.8% and 39.1%, respectively). All the OP metabolites were positively associated with maternal intake of fruits and vegetables. Other maternal characteristics related to the OPs were body mass index (BMI) before pregnancy and smoking habit during pregnancy. Women with lower BMI and those who did not smoke presented higher OP concentrations. Moreover, mothers who had a yard or garden with plants at home or who lived in an urban area were also more exposed to OPs.

**Conclusions:**

The OP detection frequencies and the concentrations observed in our study population were low, compared with most of the previously published studies. Given the high vulnerability of the fetus to neurotoxicant exposure, further research on the determinants of the body burden of OPs during pregnancy would be necessary. The knowledge gained from such studies would enhance the effectiveness of public health control and future recommendations in order to reduce the risk to both the health of pregnant women and the health and development of their children.

**Electronic supplementary material:**

The online version of this article (doi:10.1186/s12940-017-0255-z) contains supplementary material, which is available to authorized users.

## Background

Pesticides are widely used in the agricultural areas of Spain. The sales of these products over the last decade have exceeded 600 million euros annually, thus accounting for 10% of the total sales in Europe [1]. The Valencia Region is the second largest agricultural area in Spain and one of the largest pesticide users in this country. This region was responsible for more than 12% of the total national pesticide consumption in 2009 [2]. The organophosphate pesticides (OPs) are the most widely used active substance in insecticides, followed by pyrethroids and carbamates [3]. As a result of their massive use, especially chlorpyrifos, OPs spread through the environment and contaminate water, soil, and atmosphere, resulting in a potential risk to humans and the environment [4].

Pesticides are also used in domestic settings, pyrethroids being the most common active ingredient used in residential insecticides [5]. However, some OPs, such as chlorpyrifos, were commonly found in the composition of domestic insecticides in Spain [6]. As of 2008, the use of chlorpyrifos as a domestic pesticide was phased out in the EU (Directive 98/8/CE).

Prenatal exposure to OPs was first described in three birth cohort studies conducted in USA. Chlorpyrifos was detected in cord blood samples from newborns participating in the Columbia Center for Children’s Environmental Health in New York, showing a high correlation with maternal blood, which may indicate that this pesticide crosses the placenta barrier [7]. Urinary levels of some OP metabolites have also been measured among pregnant women from the Children’s Environmental Health Study in New York [8] and from the Health Assessment of Mothers and Children of Salinas (CHAMACOS study), an agricultural area in California [9]. These latter studies found detectable levels of different OP metabolites in nearly all urine samples.

There is increasing public concern about the effects associated to the exposure to pesticides during early development [10]. Fetuses and children are especially more vulnerable to exposure to environmental pollutants in comparison to adults, since their organs and systems are still developing and their detoxification mechanisms are not yet fully mature [11]. There is increasing evidence of a relationship between prenatal exposure to OPs and child neurodevelopment [12]. In addition, OPs have also been linked in epidemiology studies to shorter time of gestation [13, 14], low birth weight [15, 16], increased child blood pressure [17], respiratory outcomes [18], obesity and diabetes [19, 20]. To date, very few longitudinal studies have investigated factors associated with pesticide exposure in pregnant women [8, 21–24].

The aim of this study is to describe prenatal exposure to OPs and evaluate the associated factors in pregnant women participating in a birth cohort study located in a Spanish area with intense agricultural activity. Studies on pesticide exposure determinants are needed to identify sources and pathways of pesticide exposure and to contribute to policies aimed at reducing exposure.

## Methods

### Study population

This study is framed within the INfancia y Medio Ambiente (INMA) Project (Environment and Childhood), the aim of which is to investigate the effects of environmental exposure, diet, and genetics on fetal and child development in a cohort of pregnant women and their offspring in different regions of Spain (http://www.proyectoinma.org/). The study protocol has been reported elsewhere [25]. Briefly, pregnant women were recruited at the beginning of their pregnancy (10–13 weeks of gestation) in the region of Valencia (2003–2005, *n* = 855). These women were followed up until the third trimester of pregnancy (*n* = 794). The final study population consisted of 573 pregnant women with complete data on OP exposure. The main reason for the decrease in the study population was the unavailability of urine samples for OP measurement (*n* = 122) and the limited resources for the analysis of OPs in all available samples (*n* = 99).

### Analysis of OP metabolites in urine

Urine samples were collected at the third trimester of pregnancy (mean ± sd: 32.2 ± 1.4 weeks of gestation). Samples were separated into aliquots of 10 mL and then frozen at −20 °C until analysis in the Public Health Laboratory of Valencia. One aliquot was used for the analytical determination of metabolites in urine by using an ultra-high pressure liquid chromatography coupled with high resolution mass spectrometry method (UPLC-HRMS) [26]. Briefly, metabolites from hydrolyzed urine were extracted by liquid-liquid extraction with 10 ml of acetonitrile followed by a QuEChERS extraction [27]. The acetonitrile layer obtained was evaporated to dryness, dissolved in 200 μL of methanol: water (10/90, *v*/v) containing 0.1% of acetic acid, ultra-centrifuged (11,000 rpm, 3 min and 10 °C) and transferred into an injection vial for analysis on an Accela liquid chromatography UHPLC system from ThermoFisher Scientific (Bremen, Germany). Separation was performed by using a Hypersil Gold column (100 × 116 2.1 mm, 1.9 μ) with a flow rate of 400 μL min^−1^ and an injection volume of 10 μL. Mobile phase components were 0.1% acetic acid aqueous solution (A) and methanol containing 0.1% acetic acid (B). Targeted mass analysis was performed on an Orbitrap mass spectrometer Exactive™ analyzer (Thermo Scientific, Bremen, Germany) operating in both positive and negative ESI modes. The ion source parameters were 3.5 kV (positive mode) and 2.5 kV (negative mode); sheath gas flow-rate: 55; auxiliary gas flow-rate: 10; skimmer voltage: 23 V; heater temperature: 300 °C; capillary temperature: 150 °C; capillary voltage: 45 V, and tube lens voltage: 120 V. The system operated at a resolving power of 50,000 (250 ms). Each metabolite was identified and confirmed following the criteria of relative retention time (RRT), mass tolerance value of the molecular ion <5 ppm, isotopic pattern, and fragment ions.

The analysis included four non-specific metabolites of exposure to OPs (Table 1): diethyl phosphate (DEP), diethyl thiophosphate (DETP), dimethyl thiophosphate (DMTP), dimethyl dithiophosphate (DMDTP), and the four specific metabolites: 2-diethylamino-6-methyl-4-pyrimidinol (DEAMPY), 2-isopropyl-4-methyl-6-hydroxypyrimidine (IMPY), para-nitrophenol (PNP), and 3,5,6-trichloro-2-pyridinol (TCPY). Sum (molar) variables were also calculated: sumDEP: DEP + DETP, sumDMP: DMTP + DMDTP, sumDAP: sumDEP + sumDMP, and sumOPs: DEAMPY + IMPY + PNP + TCPY.

**Table 1:** OP metabolites analyzed and possible precursor compounds

Possible precursor compound	Metabolite	Acronym	LOD (μg/L)
Chlorethoxyphos, chlorpyrifos, coumaphos, diazinon, disulfoton, ethion, parathion, phorate, phosalone, sulfotep, terbufos	Diethyl phosphate	DEP^a^	10
	Diethyl thiophosphate	DETP^a^	3.2
Azinphos-methyl, dichlorvos, dicrotophos, dimethoate, fenitrothion, fenthion, malathion, methyl parathion, trichlorfon, chlorpyrifos-methyl, methidathion, mevinphos, oxydemeton-methyl, phosmet, primiphos-methyl, temephos, tetrachlorvinphos, isazofos-methyl, naled	Dimethyl thiophosphate	DMTP^a^	1.6
	Dimethyl dithiophosphate	DMDTP^a^	1.6
Chlorpyrifos, chlorpyrifos-methyl	3,5,6-trichloro-2-pyridinol	TCPY^b^	0.8
Parathion, methyl parathion	p-nitrophenol	PNP^b^	0.8
Pirimiphos-methyl	2-diethylamino-6-methyl-4-pyrimidinol	DEAMPY^b^	1.6
Diazinon	2-isopropyl-4-methyl-6-hydroxypyrimidine	IMPY^b^	1.6

*LOD* Limit of determination

^a^Non-specific compound

^b^Specific compound

The analytical suitability of the method was checked each working day through the analysis of the quality controls previously described by Roca et al. [26]. In accordance with this quality procedure, in each analytical batch, various quality control samples (QC) were prepared by spiking blank urine samples at LoQ, QCinter, and QChigh levels to calculate the extraction efficiency and ensure a good quantification of real samples. The QCs were subjected to the same extraction and analysis procedures as real samples and calibration curve points.

OP concentrations were expressed in μg/L and in μg/g of creatinine in order to correct for urinary dilution. Creatinine was determined by the Jaffé method (kinetic with target measurement, compensated method) with Roche reagents in a Hitachi 911 auto-analyzer.

### Covariates

The women filled in two questionnaires during pregnancy, one at the first trimester (mean ± sd: 12.1 ± 1.5 weeks of gestation) and the other at the third trimester (mean ± sd: 31.8 ± 1.7 weeks of gestation). The questionnaires were administered by trained interviewers and focused on sociodemographic, environmental, and lifestyle information during pregnancy. The maternal covariates obtained were maternal age (years), country of birth (Spain, other), educational level (up to primary, secondary, university), body mass index before pregnancy (BMI, kg/m^2^), parity (0, 1, >1), employment status during pregnancy (employed, not employed), smoking habit during pregnancy (smoker, non-smoker), season of sampling (winter, spring, summer, fall), area of residence (urban, metropolitan, semi-urban, rural), use of indoor pesticides (no, yes), yard with plants at home (no, yes), outdoor pesticides application (no, yes), residence near fields or greenhouses (no, residence near fields, residence near fields sprayed with pesticides). We also obtained information about the paternal or maternal occupation and identified those related to the use of pesticides (agriculture, gardening).

We defined social class based on the maternal or paternal occupation during pregnancy with the highest social class, according to a widely used Spanish adaptation of the coding system of the International Standard Classification of Occupations approved in 1988 (ISCO88) [28]. Class I + II included managerial jobs, senior technical staff, and commercial managers; class III included skilled non-manual workers; and class IV + V, manual workers.

The Valencia Region was divided into four sub-areas according to the population density and land uses: urban, metropolitan, semi-urban, and rural.

Dietary information was collected by using a validated semi-quantitative food frequency questionnaire (FFQ) of 101 food items [29]. The FFQ was administered during the third trimester of pregnancy (the same time point as the general questionnaire) and covered average intakes from the previous 3 months. Information about the intake of different fruits and vegetables was obtained and grouped as follows (expressed as daily servings): citrus fruits (orange and orange juice), stone fruits (prune, peach, nectarine, and apricot), kiwis, apples and pears, bananas, watermelon, fruit vegetables (courgette, aubergine, cucumber, tomato, and pepper), green vegetables, (lettuce, spinach, chard, and cabbage), other vegetables (green beans, onion, carrot, and pumpkin). Total fruit and vegetable intakes were also calculated.

### Statistical analysis

Maximum likelihood estimation of censored linear regression models was used to estimate the geometric mean (GM) and 95% confidence intervals (95%CI) for OP metabolites [30, 31]. The compounds were log-transformed so as to approach normality. This scheme also applies to molar summed variables. The OP metabolite concentrations were expressed as μg/g of creatinine, as μg/L, and as molar (nm/g of creatinine and nm/L). All individual compounds were included in the definition of sum variables, although, when studied separately, we only considered those with a detection frequency > 30%.

The association between OP metabolite concentrations and the sociodemographic, environmental, and dietary characteristics was evaluated by multivariable interval censored regression models in order to deal with values below the LOD. These models were built using a backward elimination procedure: first, all the covariates related to the OP metabolite concentrations at the univariate level (*p* < 0.1) were included in the initial models. Then, those variables not associated with the OP metabolite concentrations in the multivariable model using the Likelihood Ratio Test (*p* > 0.1) were sequentially excluded. For comparability purposes, final models were adjusted for all the covariates that were retained for at least one of the compounds. For this analysis the unadjusted OP concentrations were used including creatinine as a covariate.

In a further analysis, total fruit and vegetable intake variables were replaced by the subgroup dietary variables (citrus fruits, stone fruits, kiwis, apples and pears, bananas, watermelon, fruit vegetables, green vegetables, other vegetables) in order to evaluate their influence on OP metabolite concentrations.

## Results

Sociodemographic, environmental, and dietary characteristics of the study population are shown in Table 2. Nearly all the participants were Spaniards, 25% of them had finished university studies, around 80% worked during pregnancy, and nearly half of them belonged to the lowest social class. More than 60% of the women used domestic pesticides at home during pregnancy. Only 16 of the fathers and none of the mothers had an occupation related with the use of pesticides (agriculture or gardening).

**Table 2:** Sociodemographic, environmental, and dietary characteristics of the study population, INMA-Valencia, Spain, 2003–2006

		Study population (*n* = 573)		Population not included (*n* = 221)		*P*-value^1^
		N	%	N	%	
Maternal age (years)	<25	60	10.5	31	14	0.216
	25–29	196	34.2	85	38.5	
	30–34	230	40.1	75	33.9	
	≥35	87	15.2	30	13.6	
Country of birth	Spain	509	88.8	189	85.5	0.200
	Other	64	11.2	32	14.5	
Educational level	Up to primary	182	31.8	88	39.8	0.069
	Secondary	248	43.3	90	40.7	
	University	143	25.0	43	19.5	
Parity	0	324	56.5	114	51.6	0.002
	1	212	37.0	75	33.9	
	>1	37	6.5	32	14.5	
Employment status during pregnancy	Non-employed	101	17.6	38	17.2	0.886
	Employed	472	82.4	183	82.8	
Social class^c^	I + II (higher)	137	23.9	37	16.7	0.021
	III	161	28.1	55	24.9	
	IV + V (lower)	275	48.0	129	58.4	
Smoking habit during pregnancy	Non-smoker	447	78.0	159	74.3	0.271
	Smoker	126	22.0	55	25.7	
Season of sampling	Winter	73	12.7	42	29.8	<0.001
	Spring	106	18.5	41	29.1	
	Summer	199	34.7	21	14.9	
	Fall	195	34.0	37	26.2	
Area of residence	Urban	56	9.8	15	6.8	0.629
	Metropolitan	276	48.2	111	50.7	
	Semi-urban	206	36.0	79	36.1	
	Rural	35	6.1	14	6.4	
Use of indoor pesticides	No	212	37.1	72	33.6	0.375
	Yes	360	62.9	142	66.4	
Yard with plants at home	No	393	68.7	159	74.3	0.127
	Yes	179	31.3	55	25.7	
Outdoor pesticides application	No	487	85.1	187	87.4	0.423
	Yes	85	14.9	27	12.6	
Residence near fields or greenhouses	No	315	55.1	131	61.2	0.097
	Residence near fields	164	28.7	45	21	
	Residence near fields sprayed with pesticides	93	16.3	38	17.8	
BMI before pregnancy^a,b^		23.8	4.5	23.7	5.0	0.263
Intake of vegetables (daily servings)^a,b^		2.2	1.3	2.2	1.3	0.991
Intake of fruits (daily servings)^a,b^		2.5	1.7	2.6	1.8	0.369

^1^ *P*-value from Chi-square test

^a^mean and standard deviation

^b^ *p*-values from Mann-Whitney test

^c^Class I + II included managerial jobs, senior technical staff, and commercial managers; class III included skilled non-manual workers; and class IV + V, manual workers

Differences between the study population (*n* = 573) and the excluded women (those who arrived at the third trimester visit but for whom OP metabolite measurements are unavailable, *n* = 221) were assessed. We observed statistically significant differences according to the parity, social class, and season of sampling. Among the excluded women there was a higher proportion of parity > 1, lower social class, and urine samples taken during winter.

The frequencies of detection of OP metabolites were low, in fact only DMTP and TCPY were detected in more than 30% of the samples. The GM of the concentrations ranged from 0.02 μg/g of creatinine for IMPY and DEAMPY to 2.76 μg/g of creatinine for DEP. Regarding the sum variables, the GM of the concentrations ranged from 3.07 μg/g of creatinine for sumDEP to 16.29 μg/g of creatinine for sumDAP (Table 3).

**Table 3:** Urinary OP concentrations (creatinine adjusted and unadjusted and molar) in pregnant women from INMA-Valencia cohort (Spain, 2003–2006, *n* = 573)

			Creatinine adjusted (μg/g)					Unadjusted (μg/L)					Molar concentrations creatinine adjusted (nm/g)			Molar concentrations (nm/L)		
	N > LOD	% > LOD	Min	Max	GM	95% CI		Min	Max	GM	95% CI		GM	95% CI		GM	95% CI	
DEP	50	8.7	<LOD	66.4	1.04	0.54	2.00	<LOD	71.2	1.73	1.05	2.85	6.66	3.55	12.47	11.23	6.81	18.52
DETP	43	7.5	<LOD	56.5	0.09	0.03	0.27	<LOD	32.0	0.22	0.1	0.49	0.60	0.21	1.68	1.29	0.57	2.91
DMTP	308	53.8	<LOD	1023.0	2.76	2.23	3.42	<LOD	940.4	2.39	1.93	2.97	19.45	15.69	24.11	16.83	13.56	20.89
DMDTP	88	15.4	<LOD	189.5	0.06	0.03	0.14	<LOD	467.2	0.07	0.03	0.14	0.50	0.24	1.07	0.42	0.19	0.91
DEAMPY	32	5.6	<LOD	394.3	0.02	0.01	0.09	<LOD	293.0	0.03	0.01	0.12	0.14	0.04	0.56	0.19	0.05	0.67
IMPY	67	11.7	<LOD	787.5	0.02	0.01	0.07	<LOD	744.2	0.03	0.01	0.07	0.19	0.07	0.54	0.17	0.06	0.48
PNP	56	9.8	<LOD	28.7	0.04	0.02	0.09	<LOD	24.0	0.04	0.02	0.09	0.33	0.15	0.73	0.29	0.12	0.66
TCPY	224	39.1	<LOD	142.3	0.57	0.45	0.72	<LOD	117.3	0.49	0.39	0.62	2.88	2.29	3.62	2.47	1.95	3.13
sumDEP	76	13.3	6.6	98.2	3.07	2.21	4.27	6.6	78.0	4.72	3.76	5.93	19.57	14.11	27.13	29.78	23.71	37.42
sumDMP	314	54.8	1.6	1023.8	4.84	4.05	5.77	1.6	1015.8	4.2	3.52	5.02	32.79	27.44	39.19	28.48	23.81	34.07
sumDAP	337	58.8	8.2	1030.4	16.29	14.67	18.09	8.2	1022.4	14.64	13.25	16.18	107.25	96.45	119.27	96.15	86.87	106.42
sumOP	283	49.4	2.4	789.1	3.83	3.36	4.36	2.4	745.8	3.44	3.03	3.91	22.41	19.69	25.51	20.18	17.78	22.91

The detection frequency for the summed variables was defined as the proportion of individuals with at least one detected compound

*DEP* Diethyl phosphate; *DETP* Diethyl thiophosphate; *DMTP* Dimethyl thiophosphate; *DMDTP* Dimethyl dithiophosphate; *DEAMPY* 2-diethylamino-6-methyl-4-pyrimidinol; *IMPY* 2-isopropyl-4-methyl-6-hydroxypyrimidine; *PNP* para-nitrophenol; *TCPY* 3,5,6-trichloro-2-pyridinol; *sumDEP* DEP + DETP; *sumDMP* DMTP + DMDTP; *sumDAP* sumDEP + sumDMP; *sumOP* DEAMPY + IMPY + PNP + TCPy

The limit of determination was 10 μg/L for DEP, 2.3 μg/L for DETP, 1.6 μg/L for DMTP, 1.6 μg/L for DMDTP, 0.8 μg/L for TCPY, 0.8 μg/L for PNP, 1.6 μg/L for DEAMPY, and 1.6 μg/L for IMPY.

*LOD* limit of determination

*Min* minimum value

*Max* maximum value

*GM* geometric mean

The characteristics of the study population related to the OP metabolite concentrations can be found in Table 4. Smoking habit was significantly statistically associated with DMTP concentrations, women who smoked during pregnancy being the ones who presented lower levels. The pattern with the other compounds was similar but not significant. We observed that women who smoked during pregnancy consumed less fruit (mean = 2.24; standard deviation [sd] = 1.29 daily servings) than non-smoker women (mean = 2.58; sd = 1.62 daily servings, *p*-value Mann Whitney test =0.001). Smokers also consumed fewer vegetables than non-smokers (mean = 2.22; sd = 1.42 vs. mean = 2.24; sd = 1.29 daily servings), but the differences were not statistically significant (Additional file 1: Table S1).

**Table 4:** Sociodemographic, environmental, and dietary characteristics associated with the OP concentrations. INMA-Valencia cohort (Spain, 2003–2006)^a^

		DMTP			TCPY			sumDEP			sumDMP			sumDAP			sumOP		
		beta	95% CI		beta	95% CI		beta	95% CI		beta	95% CI		beta	95% CI		beta	95% CI	
Smoking habit	No																		
	Yes	−1.22**	−1.93	−0.51	−0.13	−0.78	0.53	0.19	−0.19	0.56	−0.23	−0.76	0.31	−0.53	−0.87	−0.19	−0.02	−0.42	0.39
Level of education	Primary																		
	Secondary	−0.40	−1.05	0.26	0.36	−0.27	0.99	0.00	−0.45	0.45	−0.23	−0.76	0.31	−0.07	−0.39	0.24	0.38*	−0.01	0.77
	University	0.27	−0.48	1.02	0.36	−0.38	1.09	0.10	−0.29	0.49	0.31	−0.30	0.93	0.13	−0.24	0.49	0.25	−0.21	0.71
Season of sampling	Winter																		
	Spring	−0.88*	−1.90	0.14	2.31**	1.15	3.46	−0.52*	−1.13	0.09	−0.55	−1.39	0.29	−0.25	−0.74	0.24	0.89**	0.26	1.53
	Summer	0.58	−0.33	1.48	3.26**	2.17	4.35	−0.31	−0.83	0.22	0.65*	−0.10	1.39	0.27	−0.16	0.71	1.15**	0.56	1.73
	Fall	0.74	−0.16	1.64	2.74**	1.64	3.83	0.27	−0.21	0.76	0.64*	−0.10	1.39	0.32	−0.11	0.76	0.74**	0.15	1.33
Zone of residence	Urban																		
	Metropolitan	−0.75	−1.69	0.18	0.15	−0.76	1.05	−0.56**	−1.06	−0.05	−0.60	−1.37	0.16	−0.37	−0.82	0.07	0.02	−0.54	0.58
	Semi-urban	−0.81	−1.80	0.18	−0.46	−1.43	0.51	−0.15	−0.66	0.36	−0.63	−1.44	0.19	−0.22	−0.70	0.25	−0.30	−0.90	0.30
	Rural	−0.58	−2.01	0.84	−0.82	−2.25	0.62	−0.33	−1.11	0.44	−0.38	−1.55	0.79	−0.15	−0.83	0.53	−0.24	−1.11	0.63
Yard with plants at home	No																		
	Yes	1.00**	0.23	1.76	0.64*	−0.10	1.38	−0.11	−0.57	0.35	0.76**	0.13	1.39	0.24	−0.13	0.61	0.16	−0.30	0.62
Application of outdoor pesticides	No																		
	Yes	−0.65	−1.62	0.31	0.02	−0.91	0.94	0.55**	0.01	1.10	−0.55	−1.34	0.25	−0.12	−0.59	0.34	0.11	−0.47	0.68
Residence near fields or greenhouses	No																		
	Residence near fields	−0.18	−0.82	0.46	−0.22	−0.84	0.40	−0.11	−0.48	0.27	−0.22	−0.75	0.30	−0.15	−0.46	0.16	0.05	−0.33	0.44
	Residence near fields sprayed with pesticides	0.26	−0.53	1.05	0.07	−0.71	0.84	0.07	−0.37	0.50	0.12	−0.53	0.77	0.07	−0.31	0.45	0.46*	−0.01	0.93
Fruit intake (daily servings)		0.26**	0.04	0.47	0.05	−0.15	0.26	0.20**	0.09	0.31	0.21**	0.03	0.38	0.14**	0.03	0.24	0.13*	0.00	0.26
Vegetables intake (daily servings)		0.26**	0.09	0.43	0.16*	0.00	0.32	0.08	−0.01	0.17	0.23**	0.10	0.37	0.12**	0.04	0.20	0.08	−0.02	0.19
BMI before pregnancy		−0.36**	−0.67	−0.05	−0.39	−0.70	−0.08	0.02	−0.15	0.19	−0.32**	−0.58	−0.07	−0.16**	−0.31	−0.01	−0.16*	−0.35	0.02

*BMI* Body mass index; *CI* Confidence intervals; *DMTP* Dimethyl thiophosphate; *TCPy* 3,5,6-trichloro-2-pyridinol; *SumDEP* Sum of diethylphosphates (DEP + DETP); *SumDMP* Sum of dimethylphosphates (DMTP + DMDTP); *SumDAP* Sum of dialkyl phosphates (SumDEP + SumDMP); *SumOP* Sum of specific compounds (DEAMPY + IMPY + PNP + TCPY)

^a^All models were adjusted by smoking habit, educational level, season of sampling, zone of residence, yard with plants at home, application of outdoor pesticides, residence near fields or greenhouses, fruit intake, vegetable intake, BMI before pregnancy, and creatinine

*p < 0.1

**p < 0.05

Women with university studies had the lowest concentrations of sumDAP. Season of urine sampling was associated with nearly all the compounds, samples taken during summer or fall being those which had the highest concentrations. Women who lived in an urban zone had higher OP metabolites, as did women who lived near fields or greenhouses sprayed with pesticides. Women who had a yard or garden with plants at home had the highest concentrations of DMTP, TCPy, and sumDMP, and women who applied outdoor pesticides had the highest concentrations of sumDEP. The intake of fruit and vegetables during the third trimester of pregnancy was positively associated with the OP metabolite concentrations, and the BMI before pregnancy was inversely associated with nearly all the compounds. We analyzed the differences in the intake of fruits and vegetables according to the maternal BMI and observed that women with BMI > 25 Kg/m^2^ consumed more vegetables (2.35 weekly servings for women with BMI = 25–30 Kg/m^2^ and 2.30 weekly servings for women with BMI ≥ 30 Kg/m^2^) than women with BMI < 25 Kg/m^2^ (2.19 weekly servings), but differences were not statistically significant (*p*-value Kruskal Wallis test = 0.416) (Additional file 1: Table S1).

Women whose partners had an occupation related with the use of pesticides had higher levels of TCPY (mean [sd] = 7.2 [15.3] μg/g of creatinine) than those whose partners did not work with pesticides (mean [sd] = 2.8 [8.1] μg/g of creatinine, *p*-value Mann Whitney test = 0.046).

**Fig. 1:**
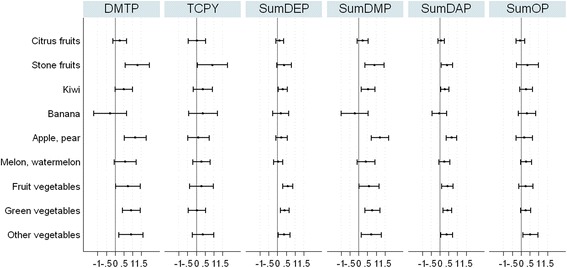
Association between the intake of different types of fruits and vegetables during pregnancy and the OP concentrations. All models were adjusted by smoking habit, educational level, season of sampling, zone of residence, yard with plants at home, application of outdoor pesticides, residence near fields or greenhouses, fruit intake, vegetable intake, body mass index before pregnancy, and creatinine.

The relationship between the intake of specific fruits and vegetables with the OP metabolite concentrations has been also evaluated (Fig. 1). The intake of green vegetables during pregnancy was significantly and positively associated with all the compounds, except for TCPy. Other groups of fruits and vegetables also associated with a considerable number of compounds (>3) were stone fruits, kiwis, apples and pears, and fruit vegetables. TCPy was the OP metabolite least associated with the intake of fruits and vegetables, and sumDAP was the OPs metabolite group associated with the intake of the highest number of different fruits and vegetables.

## Discussion

In this Spanish birth cohort study we assessed maternal exposure to OPs and evaluated the associated factors. The detection frequencies were low, DMTP and TCPY being the most frequently detected metabolites. The concentrations were positively associated with maternal intake of fruits and vegetables during pregnancy, especially the intake of green and fruit vegetables, stone fruits, kiwis, and apples and pears. Other maternal characteristics related to the OP metabolite concentrations were BMI before pregnancy and smoking habit during pregnancy. Women with lower BMI and those who did not smoke presented higher OP metabolite concentrations. Moreover, mothers who had a yard or garden with plants at home or lived in the urban area were also more exposed to OPs.

Other epidemiological studies have assessed the maternal exposure to OPs during pregnancy (Table 5). The first birth cohort studies where OP metabolites were analyzed in maternal urine were conducted in USA, one in New York City [8], and the other in an agricultural community in Salinas Valley, California [32, 33]. In both studies most of the metabolite concentrations were higher than in our study; DEP was the only one with concentrations a little higher in our study than in the USA cohorts. However, the detection frequency of this metabolite in our cohort was lower, probably due to a higher limit of detection. In our cohort the percentage of women using indoor pesticides (63%) was higher than in the New York cohort (46%), but urinary OP metabolite concentrations did not associate with this indoor use obtained by questionnaire. The possible reason could be related with differences in the time window of exposure reflected by both urinary metabolites and questionnaire. Whilst urinary biomarkers reflect acute or short-term exposure better, questionnaires may assess exposure during a period of time greater than the half-life of these substances. Another possible reason is that pyrethroids, but not OPs, were the main active substance found in the domestic insecticides [5], although some OPs, such as chlorpyrifos, diazinon, and fenitrothion were still allowed as domestic insecticides when urine samples were taken.

**Table 5:** OP concentrations and associated factors among pregnant women observed in previous studies

Study	Location	n	Metabolites measured	Levels	LOD	Factors studied	Factors associated with OPs
Berkowitz et al., 2003 [8]	New York, USA	386	TCPy	11.3 μg/g ^a^	12 μg/L	Indoor pesticides use, maternal age, ethnicity, marital status, maternal education, season	Lower/middle school (−)
			PCP	7.3 μg/g^a^	23.0 μg/L		-
Castorina et al., 2003, 2010 [32, 33]	California, USA	481	DMP	1.7 μg/L^a^	0.7 μg/L	-	-
			DMTP	6.3 μg/L^a^	0.5 μg/L		
			DMDTP	0.5 μg/L^a^	0.3 μg/L		
			DEP	1.1 μg/L^a^	0.3 μg/L		
			DETP	0.9 μg/L^a^	0.2 μg/L		
			DEDTP	0 μg/L^a, d^	0.1 μg/L		
			PNP	0.3 μg/L^a^	0.1 μg/L		
			TCPy	3.2 μg/L^a^	0.3 μg/L		
Ye et al., 2009 [34]	Oslo, Norway	110 (pooled for analysis)	DMP	12.97 μg/g (10.39 μg/L)^b^	0.1 μg/L	-	-
			DMTP	13.21 μg/g (9.91 μg/L)^b^	0.1 μg/L		
			DMDTP	0.98 μg/g (0.75 μg/L)^b^	0.1 μg/L		
			DEP	2.54 μg/g (1.94 μg/L)^b^	0.1 μg/L		
			DETP	1.51 μg/g (1.19 μg/L)^b^	0.1 μg/L		
			DEDTP	0.02 μg/g (0.18 μg/L)^b^	0.01 μg/L		
			Total DAP	31.23 μg/g (24.20 μg/L)^b^			
			TCPy	2.95 μg/g (2.33 μg/L)^b^	0.15 μg/L		
	Rotterdam (Netherlands)	100	DMP	19.78 μg/g (14.87 μg/L)^b^	0.1 μg/L		
			DMTP	19.42 μg/g (15.14 μg/L)^b^	0.1 μg/L		
			DMDTP	0.85 μg/g (0.65 μg/L)^b^	0.1 μg/L		
			DEP	5.02 μg/g (3.83 μg/L)^b^	0.1 μg/L		
			DETP	2.98 μg/g (2.49 μg/L)^b^	0.1 μg/L		
			DEDTP	0.12 μg/g (0.10 μg/L)^b^	0.01 μg/L		
			Total DAP	48.18 μg/g (37.08 μg/L)^b^			
			TCPy	4.38 μg/g (3.61 μg/L)^b^	0.15 μg/L		
	USA (NHANES)	119	DMP	2.33 μg/g (3.22 μg/L)^b^	0.5 μg/L		
			DMTP	6.47 μg/g (7.09 μg/L)^b^	0.4 μg/L		
			DMDTP	1.22 μg/g (1.50 μg/L)^b^	0.2 μg/L		
			DEP	2.36 μg/g (2.38 μg/L)^b^	0.1 μg/L		
			DETP	1.22 μg/g (1.59 μg/L)^b^	0.1 μg/L		
			DEDTP	0.18 μg/g (0.17 μg/L)^b^	0.1 μg/L		
			Total DAP	13.78 μg/g (16.96 μg/L)^b^			
			TCPy	2.59 μg/g (2.77 μg/L)^b^	0.4 μg/L		
Wang et al., 2012 [14]	Shanghai, China	187	DMP	25.75 μg/g (17.19 μg/L)^b^	Nr	-	-
			DMTP	11.99 μg/g (8.01 μg/L)^b^			
			DEP	9.03 μg/g (6.03 μg/L)^b^			
			DETP	9.45 μg/g (6.31 μg/L)^b^			
			DEDTP	P25: 0.94 μg/g			
Yolton et al., 2013 [21]	Cincinnati, USA	100	SumDEP	9.4 (8–11.1) nmol/g creatinine^c^	0.6 μg/L for DMP, 0.2 μg/L for DMTP, 0.5 μg/L for DMDTP, 0.6 μg/L for DEP, 0.4 μg/L for DETP and 0.4 μg/L for DEDTP	sex, race, maternal education, marital status, maternal employment, fresh fruit and vegetable intake, organic use	black (−), not married living alone (−), higher education (+) and veg/fruit intake (+)
			SumDMP	46.4 (40.2–53.7) nmol/g creatinine^c^			Black (−), higher education (+), not married (−), employed (+), veg/fruit intake (+), organic use (+)
			SumDAP	73.5 (64.8–83.4) nmol/g creatinine^c^			Black (−), higher education (+), not married (−), employed (+), veg/fruit intake (+), organic use (+)
Fortenberry et al., 2014 [35]	Mexico DF, Mexico	187	TCPy	1.76 (1.55. 2.02) μg/L ^c^	0.10 μg/L	-	-
Colapinto et al., 2015 [22]	Canada	1850	DMP	3.19 μg/L^b^	1.0 μg/L	Maternal tea consumption	-
			DMTP	3.29 μg/L^b^	0.6 μg/L		
			DMDTP	0.48 μg/L^b^	0.3 μg/L		
			DEP	2.46 μg/L^b^	1.0 μg/L		
			DETP	0.67 μg/L^b^	0.3 μg/L		
			DEDTP	<0.15 μg/L	0.3 μg/L		
Forde et al., 2015 [36]	Caribbean Islands	150	DEP	1.65 (1.39–1.97) μg/L^c^	1.0 μg/L	-	-
			DETP	DF < 60%	0.3 μg/L		
			DMP	1.60 (1.33–1.94) μg/L ^c^	1.0 μg/L		
Lewis et al., 2015 [23]	Puerto Rico	54	TCPy	0.4 μg/L^b^	0.1 μg/L	Sociodemographic, consumption of fruits, vegetables, and legumes in the 48 h prior to urine collection, and home pest-related issues	Decreasing age (−), grape juice intake (+), raisins (+)
			IMPY	<LOD	0.1 μg/L		Decreasing age (−), married (−), insects inside home (−)
			PNP	0.5 μg/L^b^	0.1 μg/L		Unemployed (+), collards intake (−), spinach intake (−)
			DEP	0.9 μg/L^b^	0.5 μg/L		Decreasing age (−), collards intake (+), peanuts intake (+), pesticides applied by participant (+)
			DETP	0.5 μg/L^b^	0.3 μg/L		Age (−), grape juice intake (+), raisins (+)
			DEDTP	<LOD	0.1 μg/L		Cherries intake (+), pesticides stored at home (−)
			DMP	1.4 μg/L^b^	0.5 μg/L		Married (+), grapes intake (−)
			DMTP	0.8 μg/L^b^	0.1 μg/L		Married (+)
			DMDTP	0.2 μg/L^b^	0.1 μg/L		Collards intake (+)
Sokoloff et al., 2016 [24]	Canada	1884	DMP	23 (22–24) nmol/L	1.00 μg/L	Maternal and sampling characteristics, pesticide use and dwelling characteristics, vegetables and fruit consumption, grain products consumption	
			DMTP	20 (19–22) nmol/L	0.60 μg/L		
			DMDTP	2 (2–2) nmol/L	0.30 μg/L		
			DEP	14 (14–15) nmol/L	1.00 μg/L		
			DETP	3 (3–3) nmol/L	0.60 μg/L		
			DEDTP	DF < 50%	0.30 μg/L		
			SumDMP	52 (49–55) nmol/L	0.30 μg/L		Education (+), parity (−), BMI (−), smokers (−), afternoon sample collection (+), in fasting status (−), sample collected in winter (+), consumption of sweet peppers (+), tomatoes (+), beans and dry beans (+), citrus fruits (+), apple juice (+), soy and rice beverages (+), cold cereal (+), white and whole grain bread (+)
			SumDEP	19 (18–19) nmol/L	0.30 μg/L		Education (+), household income (+), parity (−), BMI (−), smoker (−), sample collected in winter (+), consumption of citrus fruit (+), apple juice (+), pasta (−),
			SumDAP	78 (74–82) nmol/L	0.30 μg/L		

^a^Median

^b^GM

^c^GM (95%CI)

^d^No instrument response

(+) positive association with OPs

(−) negative association with OPs

Nr: not reported

DF: detection frequency

LOD: limit of dermination

Ye et al. [34] compared urinary OP metabolites among pregnant women from Norway, Netherlands, and USA. The OP metabolite concentrations were found to be higher in women from Rotterdam (Netherlands) than women from Oslo (Norway) and USA (NHANES) [34]. Regarding the comparison with our study, the metabolite concentrations observed in our study population were lower than those in the women from Norway, Netherlands, and USA. TCPy, DMTP, DEP, and DETP concentrations in our cohort were much lower than those reported for pregnant women from Shanghai (China) [14], Cincinnati (USA) [21], Mexico DF [35], and Canada [22, 24]. Similar concentrations of DEP and TCPy to those of our study were found in the Caribbean Islands [36] and Puerto Rico [23] studies, respectively, although the detection frequencies in our study were lower.

Maternal educational level was associated with TCPy concentrations in women from New York [8], women with lower/middle studies being the ones who had lower concentrations in comparison to women with a higher educational level. A similar pattern was observed in women from Cincinnati [21] and from Canada (Mirec Study) [24]: those with a higher education presented higher urinary concentrations of sumDEP and sumDMP. Other factors associated with exposure to OPs in the Cincinnati study were race, marital status, employment, and vegetable and fruit intake. Lower sumDEP, sumDMP, and sumDAP concentrations were observed among black, unmarried, and unemployed women. Similarly to our study, the intakes of vegetables and fruits were positively associated with OP metabolite concentrations. OP exposure in pregnant women from Puerto Rico was studied in relationship with their sociodemographic characteristics, consumption of specific vegetables and fruits 48 h prior to urine collection, and home pest-related issues [23]. Overall, OP metabolite concentrations were inversely associated with maternal age and positively associated with some vegetable and fruit items, such as raisins, collards, peanuts, grape juice, and cherries.

We observed that maternal BMI before pregnancy was inversely and consistently associated with nearly all the metabolites. This association has also been described in the Mirec Study [24], where BMI before pregnancy was inversely associated with both sumDMP and sumDEP. The authors commented that a possible explanation could be the awareness on the importance of good nutrition among women with BMI < 25 Kg/m^2^. In our study, we did not find statistically significant differences in the intake of vegetables and fruits according to the pre-pregnancy BMI. These results suggest that there could be another factor, maybe metabolic, that might be promoting the association between BMI and OP metabolites.

Another maternal characteristic related to the OP exposure in our study population was the place of residence. Women who lived in the urban area were more exposed to OPs. This finding was expected since Valencia is a city with a long agricultural tradition and the urban area is widely surrounded by fields with an intensive use of agriculture. In addition, women who lived near fields sprayed with pesticides presented higher sumOP concentrations. In fact, this variable has been considered a useful alternative to biomonitoring and a good proxy of OP concentrations [37].

This is one of the largest birth cohorts to evaluate prenatal exposure to OPs and to study the associated factors, and, as far as we know, the first one conducted in Spain. The longitudinal design of our study will allow evaluation of postnatal exposure and the possible associated effects on child development.

A limitation in this study could be that a single spot measurement may not adequately characterize exposure over the whole pregnancy, given that OPs have a fast clearance from the body. In fact, results from the CHAMACOS cohort showed impairment in children’s cognitive performance at 7 years old [38] associated to an average of two DAP metabolite measures during pregnancy, but not when the one-point-in-time measures were evaluated. We collected urine samples from all the INMA participants over two different periods during pregnancy, the first and third trimesters, but due to limited resources to measure OP metabolites in all the urine samples we only have one measurement. The average of two OP metabolite measurements would have probably been a more adequate proxy of prenatal exposure. Additionally, there was a long time lag between collection of samples and analysis. The low concentrations observed in this population could be related with this limitation; in fact, Hoppin et al. [39] observed that urinary TCPY concentrations decreased with the time elapsed since collection at room temperature; however, our samples were frozen at −20 °C immediately after collection and this drop in OP metabolite concentrations is expected not be so important. Our FFQ covered average intakes during the 3 months before the sampling, when the half-life of these compounds is shorter. In any case, we observed several statistical associations with the intake of some fruits and vegetables.

Another important consideration is the limitation of measuring OP metabolites instead of parent compounds. The metabolism of parent compounds results in the production of active oxon forms, responsible for the developmental neurotoxicity [40]. These oxon forms are metabolically converted to less toxic compounds (DAPs) via hydrolysis. However, the hydrolysis of parent compounds present in food and the environment also generates DAP metabolites that are not toxic when consumed [41–43]. Therefore urinary non-specific metabolites may represent exposure to parent pesticides and to preformed derivatives from food and the environment, and the measurement of DAP metabolites could overestimate the real exposure to parent compounds. In fact, Yolton et al. [21] observed that higher urinary concentrations of DE metabolites were associated with improved attention in infants. This positive association seems to be related to the DAP metabolites present in vegetables and fruits that could be acting as a proxy of the nutrients and antioxidants present in diet. Despite this limitation, DAP metabolites have been extensively used in epidemiological studies because these metabolites are common to the majority of OP pesticides, and laboratory methods are not available for pesticide-specific metabolites. Consequently, the non-specific DAPs provide valuable information about cumulative exposure to the OP class [44].

Finally, we were able to provide an estimate of the geometric mean for all metabolites using censored regression. However, this estimation could be imprecise due to the distributional assumptions of these models and the high proportion of undetected values.

## Conclusions

In conclusion, the OP metabolite detection frequencies and the concentrations observed in our study population were low, compared with previously published studies. For TCPy, the metabolite of chlorpyrifos, the concentrations were low despite the fact that it was still allowed as a domestic pesticide in Spain when the urine sampling was performed. Some maternal characteristics were associated with prenatal OP exposure. More in-depth knowledge of the determinants of the body burden of OPs during pregnancy would enhance the effectiveness of public health control and future recommendations for decreasing the levels of these compounds, thus reducing the risk for the health of pregnant women and the health and development of their children.

## Additional file

Additional file 1: Table S1. Intake of fruit and vegetables according to the maternal sociodemographic and life style characteristics. (DOC 49 kb)

## Abbreviations

BMI: Body mass index before pregnancy
DEAMPY: 2-diethylamino-6-methyl-4-pyrimidinol
DEP: Diethyl phosphate
DETP: Diethyl thiophosphate
DMDTP: Dimethyl dithiophosphate
DMTP: Dimethyl thiophosphate
FFQ: Food frequency questionnaire
GM: Geometric mean
IMPY: 2-isopropyl-4-methyl-6-hydroxypyrimidine
INMA: INfancia y Medio Ambiente
LOD: Limit of determination
OPs: Organophosphate pesticides
PBA: Phenoxybenzoic acid
PCP: Pentachlorophenol
PNP: Para-nitrophenol
sumDAP: sumDEP + sumDMP
sumDEP: DEP + DETP
sumDMP: DMTP + DMDTP
sumOP: DEAMPY + IMPY + PNP + TCPY
TCPy: 3,5,6-trichloro-2-pyridinol
UPLC-HRMS: High resolution mass spectrometry
