# Supplementary material for: Distributions and determinants of urinary biomarkers of organophosphate pesticide exposure in a prospective Spanish birth cohort study
Source: Environ Health. 2017 May 17;16:46. doi: 10.1186/s12940-017-0255-z (PMC5436449; doi:10.1186/s12940-017-0255-z)
Supplement: Additional file 1: Table S1. — Intake of fruit and vegetables according to the maternal sociodemographic and life style characteristics. (DOC 49 kb) [file 12940_2017_255_MOESM1_ESM.doc]

**Table S1:** Intake of fruit and vegetables according to the maternal sociodemographic and life style characteristics.

|  |  | **Vegetables** | | | **Fruits** | | |
| --- | --- | --- | --- | --- | --- | --- | --- |
|  |  | Mean | Sd | p-value | Mean | Sd | p-value |
| Smoking habit during pregnancy | Non-smoker | 2.24 | 1.29 | 0.655 | 2.58 | 1.62 | 0.001 |
|  | Smoker | 2.22 | 1.42 |  | 2.24 | 1.85 |  |
| Educational level | Up to primary | 2.26 | 1.51 | 0.745 | 2.66 | 1.85 | 0.048 |
|  | Secondary | 2.21 | 1.26 |  | 2.35 | 1.64 |  |
|  | University | 2.24 | 1.14 |  | 2.57 | 1.48 |  |
| Area of residence | Urban | 2.33 | 1.35 | 0.312 | 2.70 | 1.42 | 0.214 |
|  | Metropolitan | 2.14 | 1.21 |  | 2.59 | 1.87 |  |
|  | Semi-urban | 2.29 | 1.47 |  | 2.32 | 1.42 |  |
|  | Rural | 2.46 | 1.08 |  | 2.67 | 1.78 |  |
| Yard with plants at home | No | 2.19 | 1.30 | 0.305 | 2.51 | 1.75 | 0.644 |
|  | Yes | 2.32 | 1.35 |  | 2.48 | 1.49 |  |
| Outdoor pesticides application | No | 2.20 | 1.30 | 0.221 | 2.51 | 1.72 | 0.708 |
|  | Yes | 2.40 | 1.40 |  | 2.48 | 1.42 |  |
| Residence near fields or greenhouses | No | 2.15 | 1.26 |  | 2.53 | 1.69 |  |
|  | Residence near fields | 2.43 | 1.49 | 0.226 | 2.50 | 1.80 | 0.734 |
|  | Residence near fields sprayed with pesticides | 2.15 | 1.11 |  | 2.41 | 1.39 |  |
| BMI before pregnancy (Kg/m^2^) | <25 | 2.19 | 1.27 | 0.416 | 2.53 | 1.71 | 0.288 |
|  | 25-30 | 2.35 | 1.50 |  | 2.30 | 1.43 |  |
|  | >=30 | 2.30 | 1.22 |  | 2.71 | 1.90 |  |

Vegetables and fruit intake expressed as weekly servings

Sd: standard deviation

BMI: Body mass index

p-values calculated with the Mann Whitney test for the 2-category variables and with the Kruskal Wallis test for variables with >2 categories
